# Atypical Autism: Causes, Diagnosis and Support

**DOI:** 10.3390/medicina60071163

**Published:** 2024-07-18

**Authors:** Rekha Jagadapillai

**Affiliations:** Department of Pediatrics, Pediatric Research Institute, University of Louisville, Louisville, KY 40202, USA; rekha.jagadapillai@louisville.edu

Autism spectrum disorder (ASD) is a group of neurobehavioral disorders defined by persistent deficits in social communication and social interactions with repetitive behaviors, and it is typically diagnosed within the first three years of life [1]. The Center for Disease Control estimates that 1 in 59 eight-year-old children is diagnosed with ASD [2]. The pathogenesis of ASD is not fully elucidated and is likely to have multiple etiologies with heterogeneous clinical presentations. ASD without a defined specific cause is referred to as idiopathic ASD and constitutes the majority of cases. Several environmental factors may contribute to idiopathic ASD, such as maternal immune activation or bacterial infection and prenatal exposure to toxins [3,4]. De novo mutations are among the known genetic causes of ASD, having been identified in approximately 20–30% of cases. Three kinds of genetic abnormalities associated with ASD have been defined: (1) single-gene mutations, such as those found in *SHANK3*, *FMR1*, or *MECP2*; (2) copy number variations (CNVs) including duplications, large deletions, inversions, and translocations of chromosomes; and (3) polygenic risk factors due to the accumulation of common variants, each contributing to a portion of the risk.

Atypical autism, otherwise known as pervasive developmental disorder not otherwise specified (PDD-NOS), is used to classify individuals who show mild-to-moderate symptoms of ASD that are not enough for a diagnosis. The process of identifying a common profile of the disease and finding causative factors, diagnostic criteria, and potential treatments has been complicated by the genetic and molecular heterogeneity of ASD.

A diagnosis of autism is also a life-changing experience for parents and caregivers and involves the imposition of management, socio-economic, and emotional burdens. Families are disposed to experience long-term challenges in various aspects of their daily lives since a large percentage of autistic individuals present cognitive impairments that limit their ability to lead independent lives [5,6]. It cannot be ignored that, because of the lifelong nature of the condition and the variability in individuals with ASD, caretakers and families are likely to endure challenges in different phases of their daily lives. It is important to expand our understanding of current research in the field of autism, as well as its management and support strategies, in order to care for individuals with ASD. This Special Issue of *Medicina*, titled “Atypical Autism: Causes, Diagnosis and Support” includes research articles, review articles, and case reports offering significant current information on ASD research, support, and different aspects of management strategies for individuals with ASD.

There has been an increasing trend of autism prevalence globally despite the development of various support and management strategies. In their review article, Mondal et al. describe how using complementary and alternative strategies to manage ASD is critical [7]. The authors focus on non-pharmacological interventions including diets, supplements, antioxidants, hormones, vitamins, and minerals to manage ASD, additionally discussing the benefits of herbal medicines, cannabidiols, gut microbiomes, etc. The authors also describe a variety of dietary changes for ASD management, including the elimination of certain food from the diet, although this needs to be closely controlled, as this can prevent individuals with ASD from obtaining adequate nutrients. For example, casein and gluten cause immune responses for some individuals, as these are known to cause gut inflammation. The authors also emphasize that consuming a specific carbohydrate diet or ketogenic diet has potential beneficial effects for some individuals with ASD. Mondal et al. also shed light on findings on the consumption of nutritional supplements such as omega-3 fatty acids, zinc, vitamins, iron, magnesium, selenium, etc., which have been shown to have beneficial effects for individuals with ASD. However, these supplements, as well as their required dosage, should only be taken after consulting with a physician. These authors illustrate the beneficial effects of antioxidants for individuals with ASD and provide a brief account on some antioxidants such as curcumin, resveratrol, naringenin, sulforaphane, and luteolin in their review report. They also discuss the importance of exploring hormonal therapies and herbal medicine, as these may have some beneficial effects on some individuals with ASD to manage some of their symptoms. Cannabidiol for the management of ASD is a widely discussed topic; however, while its beneficial effects are promising, more research and studies need to be conducted in order to better understand it and its mechanisms in a wide range of use for individuals with ASD.

In their brief report, Reis et al. examined the impact of both parental presence and the use of antipsychotic drugs on social interaction and apathy among individuals with ASD [8]. They found that children on risperidone scored higher in social communication but demonstrated differences in various social interactions, particularly regarding stereotyped behaviors, although the authors could not state whether these differences were caused by drug usage or if the patients used antipsychotic drugs specifically because of these behaviors. They also reported the presence of married parents as an another statistically significant variant, reporting that children lacking the presence of married parents demonstrated more stereotypical behavior on average when compared to children raised with the presence of married parents. The authors point out that these findings might indicate the significance of the presence of both parents for the social development of a child with autism.

In their research article, Yildirin et al. reported on whether the serum levels of kynurenine pathway (KP) products and interleukin-6 (IL-6)-activating indolamine 2–3 dioxygenase (IDO) were different between children with and without ASD [9]. They also aimed to discover whether there were any differences in the biochemical parameters of children with regressive-type and non-regressive-type ASD. The results of their study showed that kynurenic acid, kynurenine, and IL-6 were higher in children with ASD when compared to the control group, whereas there were no significant differences in quinolinic acid between the groups. They also found no significant differences between the children with and without regressive-type ASD regarding kynurenic acid, kynurenine, IL-6, and quinolinic acid serum levels. The authors argue that these biomarkers should be measured in all ASD cases, and these can serve as an important diagnostic tool for ASD.

In their review, Papadopoulos et al. aimed to investigate the plausible effect of bilingualism on the linguistic skills of children with ASD living in Greece [10]. On the basis of selected articles from peer-reviewed journals, the authors’ scoping review was undertaken by addressing the following research questions: “What are the effects of bilingualism in ASD Greek-speaking children as regards their language skills? Will bilingual ASD children differ from monolingual ASD children in the Greek context?”. Based on their analysis, the authors’ review highlights two important findings. In specific language tasks, bilingual children with ASD might have advantages over monolingual children with ASD, but evidence for this was not adequate enough to be generalizable in broader Greek or international contexts. Moreover, their findings indicated that bilingualism did not hinder the language skills of children with ASD in the Greek sample studied. The authors thus point out that it will be beneficial to conduct studies on the cognitive abilities of bilingual children with ASD and the potential effect of bilingualism on language skills.

In the case report “Combined aCGH and Exome Sequencing Analysis Improves Autism Spectrum Disorders Diagnosis”, Ranieri et al. report a case of a child suspected as having ASD, who was examined by utilizing a genomic high-resolution method [11]. In their report, they showed that, via array comparative genomic hybridization (a-CGH) and exome sequencing, a paternally inherited duplication of chromosome 7 was identified, implying the involvement of the CNTNAP2 gene, which is a gene linked to brain development and cognition. Furthermore, they reported that exome sequencing also identified five potentially clinically relevant variants. Since ASD has complex origins, involving behavioral and genetic traits, additional explorations are required to discover the potential role of environmental and epigenetic characteristics. Through this case report, the authors highlight the need for using vast genomic analyses strategies to clarify the bases of conditions such as ASD.

In summary, the articles in this Special Issue shed light on current scientific research in the field of autism, as well as the importance of caregiving, management, diagnosis, and support for individuals with ASD. Hopefully, this collection of articles will be helpful for the scientific community to further more research in this area and afford progress in the diagnostic and management aspects of ASD.

As Academic Editor, I would like to thank all the authors and reviewers who contributed to this Special Issue. Additionally, I would also like to thank the *Medicina* journal team for their support, which lead to the success of this Special Issue.

## References

[1] Pasciuto E., Borrie S.C., Kanellopoulos A.K., Santos A.R., Cappuyns E., D’Andrea L., Pacini L., Bagni C. (2015). Autism Spectrum Disorders: Translating human deficits into mouse Behavior. Neurobiol. Learn. Mem..

[2] Baio J., Wiggins L., Christensen D.L., Maenner M.J., Daniels J., Warren Z., Kurzius-Spencer M., Zahorodny W., Robinson Rosenberg C., White T. (2018). Prevalence of Autism Spectrum Disorder Among Children Aged 8 Years—Autism and Developmental Disabilities Monitoring Network, 11 Sites, United States, 2014. MMWR Surveill. Summ..

[3] Ergaz Z., Weinstein-Fudim L., Ornoy A. (2016). Genetic and non-genetic animal models for autism spectrum disorders (ASD). Reprod. Toxicol..

[4] Varghese M., Keshav N., Jacot-Descombes S., Warda T., Wicinski B., Dickstein D.L., Harony-Nicolas H., Rubeis S.D., Drapeau E., Buxbaum J.D. (2017). Autism spectrum disorder: Neuropathology and animal models. Acta Neuropathol..

[5] Curley K., Colman R., Rushforth A., Kotera Y. (2023). Stress Reduction Interventions for Parents of Children with Autism 1025 Spectrum Disorder: A Focused Literature Review. Youth.

[6] Volkmar F., Pauls D. (2003). Autism. Lancet.

[7] Mondal A., Sharma R., Abiha U., Ahmad F., Karan A., Jayaraj R.L., Sundar V. (2023). A Spectrum of Solutions: Unveiling Non-Pharmacological Approaches to Manage Autism Spectrum Disorder. Medicina.

[8] Reis R.d.C., Souza I.N.B., Dias M.C.R., Mendes C.M.d.M., Almeida K.J. (2023). Association of Parental Support with Reduced Stereotypy in Children with Autism Spectrum Disorder: A Cross-Sectional Study. Medicina.

[9] Yildirim V., Simsek S., Cetin I., Dokuyucu R. (2023). Kynurenine, Kynurenic Acid, Quinolinic Acid and Interleukin-6 Levels in the Serum of Patients with Autism Spectrum Disorder. Medicina.

[10] Papadopoulos A., Prentza A., Voniati L., Tafiadis D., Trimmis N., Plotas P. (2024). Assessing the Impact of Bilingualism on the Linguistic Skills of Children with Autism Spectrum Disorder (ASD) in Greece: A Scoping Review. Medicina.

[11] Ranieri A., Veneruso I., La Monica I., Pascale M.G., Pastore L., D’Argenio V., Lombardo B. (2022). Combined aCGH and Exome Sequencing Analysis Improves Autism Spectrum Disorders Diagnosis: A Case Report. Medicina.

